# A robust model of natural hepatitis C infection using hepatocyte-like cells derived from human induced pluripotent stem cells as a long-term host

**DOI:** 10.1186/s12985-016-0519-1

**Published:** 2016-04-05

**Authors:** Khanit Sa-ngiamsuntorn, Adisak Wongkajornsilp, Phetcharat Phanthong, Suparerk Borwornpinyo, Narisorn Kitiyanant, Wasun Chantratita, Suradej Hongeng

**Affiliations:** Department of Biochemistry, Faculty of Pharmacy, Mahidol University, Bangkok, 10400 Thailand; Department of Pharmacology, Faculty of Medicine Siriraj Hospital, Mahidol University, 2 Wanglang Road, Bangkoknoi, Bangkok, 10700 Thailand; Stem Cell Research Group, Institute of Molecular Biosciences, Mahidol University, Nakhon Pathom, 73170 Thailand; Department of Biotechnology, Faculty of Science, Mahidol University, Bangkok, 10400 Thailand; Department of Pathology, Faculty of Medicine Ramathibodi Hospital, Mahidol University, Bangkok, 10400 Thailand; Department of Pediatrics, Faculty of Medicine Ramathibodi Hospital, Mahidol University, 270 Rama VI Road, Ratchatewi, Bangkok, 10400 Thailand

**Keywords:** Induced pluripotent stem cell, iPSC, Hepatocyte, Hepatocyte-like cell, Differentiation, Cytochrome P450, Drug metabolism, Hepatitis C virus, HCV, JFH-1, Cell culture model

## Abstract

**Background:**

Hepatitis C virus (HCV) could induce chronic liver diseases and hepatocellular carcinoma in human. The use of primary human hepatocyte as a viral host is restrained with the scarcity of tissue supply. A culture model restricted to HCV genotype 2a (JFH-1) has been established using Huh7-derived hepatocyte. Other genotypes including the wild-type virus could not propagate in Huh7, Huh7.5 and Huh7.5.1 cells.

**Methods:**

Functional hepatocyte-like cells (HLCs) were developed from normal human iPS cells as a host for HCV infection. Mature HLCs were identified for selective hepatocyte markers, CYP450s, HCV associated receptors and HCV essential host factors. HLCs were either transfected with JFH-1 HCV RNA or infected with HCV particles derived from patient serum. The enhancing effect of α-tocopherol and the inhibitory effects of INF-α, ribavirin and sofosbuvir to HCV infection were studied. The HCV viral load and HCV RNA were assayed for the infection efficiency.

**Results:**

The fully-developed HLCs expressed phase I, II, and III drug-metabolizing enzymes, HCV associated receptors (claudin-1, occludin, CD81, ApoE, ApoB, LDL-R) and HCV essential host factors (miR-122 and SEC14L2) comparable to the primary human hepatocyte. SEC14L2, an α-tocopherol transfer protein, was expressed in HLCs, but not in Huh7 cell, had been implicated in effective HCVser infection. The HLCs permitted not only the replication of HCV RNA, but also the production of HCV particles (HCVcc) released to the culture media. HLCs drove higher propagation of HCVcc derived from JFH-1 than did the classical host Huh7 cells. HLCs infected with either JFH-1 or wild-type HCV expressed HCV core antigen, NS5A, NS5B, NS3 and HCV negative-stand RNA. HLCs allowed entire HCV life cycle derived from either JFH-1, HCVcc or wild-type HCV (genotype 1a, 1b, 3a, 3b, 6f and 6n). Further increasing the HCVser infection in HLCs was achieved by incubating cell with α-tocopherol. The supernatant from infected HLCs could infect both naïve HLC and Huh7 cell. Treating infected HLC with INF-α and ribavirin decreased HCV RNA in both the cellular fraction and the culture medium. The HLCs reacted to HCVcc or wild-type HCV infection by upregulating TNF-α, IL-28B and IL-29.

**Conclusions:**

This robust cell culture model for serum-derived HCV using HLCs as host cells provides a remarkable system for investigating HCV life cycle, HCV-associated hepatocellular carcinoma development and the screening for new anti HCV drugs.

**Electronic supplementary material:**

The online version of this article (doi:10.1186/s12985-016-0519-1) contains supplementary material, which is available to authorized users.

## Background

Human primary hepatocytes should have served as ideal host cells for liver-targeting pathogens, e.g., malarial parasite, hepatitis B, hepatitis C and dengue viruses. The shortcoming in the procurement of these cells and their limited life span have limited the study of host-pathogen relationships and their sequential developments, e.g., xenobiotic screening, and biotransformation. A cellular substitute that closely mimics primary hepatocyte but circumvents these shortcoming would greatly extend our understanding of host-pathogen interactions and the corresponding treatment strategies.

Hepatitis C virus (HCV) [1] is a single positive-stranded RNA virus in the genus of *Hepacivirus* and *Flaviviridae* family [2]. Chronic HCV infection led to cirrhosis and hepatocellular carcinoma [3]. The self-renewal capability of liver cell was disrupted that eventually required liver transplantation or bio-artificial liver device [4]. Liver transplantation were faced with serious and faster HCV reinfection to the graft [5, 6]. An alternative for liver transplant was the hepatocyte transplant that might alleviate the demand of donor organs [7]. Direct-acting antivirals (DAA) targeting HCV enzymes was hampered with eventual drug resistance [8–10]. The development of suitable culture models for HCV is critical for designing efficacious anti-HCV strategies.

The studies on HCV life cycle relied heavily on human hepatocellular carcinoma cells (Huh7 and their derivatives) [11]. HCV genotype 2a (JFH-1), but not others, could be generated from Huh7 derived cells [12, 13]. The use of hepatocellular carcinoma as cellular host could not completely mimic primary human hepatocyte. The cancer cells actively entered cellular division while the primary hepatocytes were mostly in quiescent stage [14]. Most hepatoma cell lines usually lack various functional enzymes such as CYP450s and other phase I, II and III drug metabolizing enzymes that make them not suitable for the assessment of anti-HCV drug interaction and metabolism [15, 16]. Huh7.5.1 was derived from Huh7.5 [17], which in turn was originated from Huh7 [18]. These cells carried a mutation in the retinoic-inducible gene I (RIG-I) [19]. RIG-I played a central role in viral genome recognition and host immune response. Primary human hepatocytes have been indorsed by several groups as the major host cells for HCV [20–22]. However, the handling primary human hepatocytes faced several limitations: 1) The mature hepatocytes could not be readily proliferated in culture condition; 2) The donor supply was limited; and 3) The batch to batch variation was substantial [23].

Human induced pluripotent stem (iPS) cells could be generated from somatic cell through exogenous expression of Oct4, Sox2, KLF4 and c-MYC [24, 25]. Human iPS cells actively entered cellular division and could be differentiated into hepatocyte-like cells (HLCs) [26] and others. The use of HLCs derived from either iPS or embryonic stem cells as cellular hosts for HCV were recently reported [27–30]. These differentiated cells displayed essential liver functions and achieved nearly mature hepatocytes [31], including α-fetoprotein, albumin, phase I and phase II drug metabolizing enzymes. HLCs also expressed known HCV host receptors involved in HCV entry (Claudin-1, Occludin, SR-BI, CD81) and supported complete life cycle of HCV genotype 2a up to 21 days [30]. Nevertheless, the maturity, homogeneity and long-term stability of these HLCs have not been revealed. The HLCs not only carried less CYP450 expression than that of primary hepatocytes, but retained several fetal hepatocyte markers.

In this studies, human iPS cells generated from hMSCs using polycistronic OSKM reprograming factors [32] were differentiated into HLCs. The CYP expressions were readily inducible upon the exposure to the prototypic inducers in a similar fashion to the primary hepatocytes. Since the HCV cell receptors and cell host factors were highly expressed, these HLCs were promptly taken as HCV hosts. The infected cells were studied for viral life cycle after the transfection/infection with HCVcc and HCVser. HLCs could sustain the replication of not only JFH-1 HCV but several wild-type HCV’s from patients’ sera. High HCV titers were detected in culture medium for at least 6 months. The released HCV virions could infect naïve HLCs and were susceptible to the treatment with interferon, ribavirin or sofosbuvir.

## Methods

### Generation of human induced pluripotent stem cell (hiPSC) from hMSC

The mononuclear cells were separated from bone marrow using IsoPrep (Robbins Scientific, Canada) density gradient centrifugation. Isolated cells were seeded at 2 × 10^6^ cells on T-75 cell culture flask in Minimum Essential Medium (MEM) α (Gibco Invitrogen, NY), 10 % FBS (HyClone, GE Healthcare Life Sciences, Singapore), 100 units/mL penicillin, 100 μg/mL streptomycin at 37 °C in 5 % CO_2_. Lentiviral particles were produced by co-transfection of packaging psPAX2 packaging plasmid, pMD2.G vesicular stomatitis virus G envelope, and the polycistronic plasmid encoding human OCT4, SOX2, KLF4, c-MYC and dTomato [32] using X-tremeGENE HP DNA Transfection Reagent (Roche Diagnostics, USA). hMSCs were transduced with concentrated lentivirus (MOI = 0.5). Production of dTomato was observed 48 h post-transduction. The transduced cells were seeded on MEF in hES medium to promote reprogramming process and colony formation. Reprogrammed colonies were identified with StainAlive TRA-1-60 Antibody (Stemgent, MA). Positive colonies were manually picked and passaged until stable iPS cells established.

### Maintaining and characterization of hiPSC

Human iPS cells were maintained in a feeder free condition on Geltrex-coated dish in E8 medium at 37 °C in 5 % CO_2_. The E8 medium consisted of DMEM/F12 supplemented with 64 mg/L L-ascorbic acid-2-phosphate, 14 μg/L sodium selenite, 100 μg/L FGF2, 19.4 mg/L insulin, 543 mg/L NaHCO_3_, 10.7 mg/L transferrin and 2 μg/L TGF-β1. The medium was changed daily. Human iPS cell colonies were split at a ratio of 1:8 every 5 - 7 d and passaged onto a new Geltrex-coated dish. The iPSC colonies after the tenth passage were characterized for pluripotent markers, e.g., alkaline phosphatase, OCT4, SOX2, NANOG, TRA-1-60, TRA-1-81, and SSEA4 using alkaline phosphatase ES characterization kit (MERCK Millipore, USA), fluorescent antibody staining and quantitative RT-PCR analysis.

### Teratoma formation

Approximately 2 × 10^6^ iPS cells were subcutaneously injected into nude mice (BALB/cMlac-nu). The tumor-like tissue was collected within 8–12 weeks after injection. The tissue was fixed in 10 % formalin and underwent histological processes. The tissue sections were stained with hematoxylin and eosin and examined under a light microscope for the presence of the specialized cells derived from three germ layers. All experiments performed on laboratory animals were reviewed and approved by the Animal Care and Use Committee, Institute of Molecular Biosciences, Mahidol University.

### Differentiation of hiPSC to homogeneous hepatocyte-like cell

The characterized iPS cells were taken for hepatic induction. They were seeded on Geltrex-coated 6 well plate in E8 medium until 80 % confluence and differentiated using a modified hepatic lineage development protocol [31]. Briefly, the cultured cells were maintained in endoderm differentiation medium DMEM/F12 : IMDM (1:1) (Gibco Invitrogen, NY), 100 ng/mL activin A (PeproTech, US), 10 ng/mL bFGF, 10 ng/mL BMP-4, 1 μM LY294002 and 3 μM CHIR99021 for 24 h. Cells were maintained in endoderm commitment medium DMEM/F12 : IMDM (1:1), 100 ng/mL activin A, 100 ng/mL bFGF, 10 ng/mL BMP-4 and 1 μM LY294002 for 24 h. The endoderm-like cells were further maintained in anterior definitive endoderm differentiation medium RPMI/B27 with 50 ng/mL activin A for 3 d with a daily medium replacement. The differentiated cells were maintained in hepatic lineage specification medium RPMI/B27, 20 ng/mL BMP-4 and 10 ng/mL FGF10 for 4 d with daily medium replacement. The differentiated cells were cultured in hepatocyte basal medium (HBM, Lonza, UK), 30 ng/mL oncostatin M and 50 ng/mL HGF for 10 d with medium replacement every 2 d. For functional hepatocyte induction, the cells were cultured in Williams' Media E (Invitrogen, MD, 1 % DMSO for 3 d.

### Periodic acid-Schiff staining of glycogen

HLCs and hMSCs were cultured on 35-mm dish for 3d. The cells were fixed in 5 % formaldehyde, 95 % ethanol for 1 min, rinsed for 1 min under running tap water, immersed in periodic acid solution for 5 min, rinsed thrice with dH_2_O, immersed in Schiff’s reagent for 15 min, and rinsed with running tap water for 5–10 min. Samples were counterstained with Mayer’s hematoxylin for 1 min, rinsed with water, and assessed under light microscope.

### Hepatocyte-specific markers and cytochrome P450 expressions

The total RNA was extracted from iPS cells, HLCs, or HepaRG (HPRGC10, Life Technologies) by illustra RNAspin Mini RNA Isolation Kit (GE Healthcare, Asia Pacific) following the manufacturer instruction. The total RNA was immediately used for reverse transcription to construct cDNA using the ImProm-II reverse transcription system (Promega, WI) following the manufacturer instruction. The hepatocyte markers and cytochrome P450 markers included albumin, α-fetoprotein, cytokeratin18, G-6-PD, HNF-4α, tyrosine aminotransferase and major 7 CYPs isozymes. All gene specific primers (Additional file 1) were designed using vector NTI version 11.5 (Invitrogen, MD). They were amplified using KAPA SYBR^®^ FAST qPCR Kits (Kapa Biosystems). Each sample was measured in triplicate. PCR amplicons were confirmed using size and melting curve analysis. Expression levels were calculated using the ΔΔCt method and normalized to the endogenous GAPDH. ΔΔCt was transformed into fold change using the formula: fold change = 2^- ΔΔCt^.

### Immunofluorescent staining

HLCs on 24-well plate were fixed with 4 % paraformaldehyde for 20 min at room temperature. The fixed cells were washed thrice with PBS, blocked and permeabilized with 0.2 % Triton X-100, 3 % BSA and 2 % normal goat serum for 1 h. Primary antibodies against pluripotent markers were TRA-1-60 (1:250, SC21705, Santa Cruz Biotechnology), SSEA4 (1:250, ab16287, Abcam), NANOG (1:250, SC33759, Santa Cruz Biotechnology), OCT4 (1:250, SC5279, Santa Cruz Biotechnology). Primary antibodies against HCV cell receptors were claudin-1 (1:100, ab15098), occluding (1:100, ab31721), CD81 (1:100, ab79559), SR-BI (1:200, ab106572) and hepatitis C core antigen (1:200, ab2740) ordered from Abcam. Primary antibodies against non-structural proteins of HCV were NS3 (1:200, SC69938), NS5A (1:200, SC52417) and NS5B (1:200, SC58146) ordered from Santa Cruz Biotechnology. The cells were incubated with the primary antibody for overnight h at 4 °C in moist chamber. After washing thrice, samples were incubated with the secondary antibody; Alexa fluor 488 conjugated goat anti-mouse IgG (1:1000, A11001) or Alexa fluor 488 conjugated goat anti-rabbit IgG (1: 1000, A11003s) or Alexa fluor 488 conjugated rabbit anti-goat IgG (1: 1000, A1178) (Life Technologies) for 1 h at 37 °C. Fibroblasts or untreated hMSCs served as negative controls. After washing thrice, the cells were counterstained with DAPI and mounted with anti-fade mounting medium in coverslip and examined under a fluorescent microscope and photographed.

### The induction of major CYP450 isozymes in HepaRG and HLCs using the enzyme inducer cocktail

The expression of CYP450 levels of hepatocyte-like cells responded to enzyme inducers was evaluated after incubate with cocktail of classical drugs [33]. HepaRG and hepatocyte-like cell derived from iPS cells were seeded on 6 well-plates for 3 d. These cells were maintained in Williams’ Media E, 10 % FBS before exposure to the inducers. Both of hepatocyte cells were incubated for 72 h with the cocktail agent: 20 μM rifampicin, 50 μM omeprazole, 1 mM phenobarbital and 88 μM ethanol. The treated cells were washed with 2–3 mL PBS, detached using 0.025 % trypsin-EDTA, and neutralized with 10 % FBS in DMEM/F12. The cell pellets from hepatocyte-like cell and HepaRG were stored at -80 °C until analysis for CYP450 gene expression.

### The detection of CYP450 activities

CYP1A1, CYP2B6, CYP2C9 and CYP3A4 enzyme activities were evaluated directly in all cultured cells (HepaRG, HLCs and primary human hepatocytes) attaching to the collagen type IV-coated 6-cm dish at a density of 10^6^ cell. All cultured cells were treated with a cocktail of 20 μM rifampicin, 50 μM omeprazole, 1 mM phenobarbital and 88 μM ethanol. All treated cells were incubated for 72 h with daily medium change. CYP450 activities were assessed based on luciferase activity using the P450-glo 1A1, 2B6, 2C9 and 3A4 assays (V8751, V8321, V8791, V9001; Promega, WI). After 3-d incubation period, cells were incubated with Williams’ Media E supplemented with 100 μM Luciferin-CEE, Luciferin-2B6, Luciferin-H for 3–4 h or 3 μM Luciferin-IPA for 30–60 min. An aliquot (50 μL) of the medium was transferred to 96-well opaque white luminometer plate (Nunc, Denmark) and luciferin detection reagent was added to each well. After sitting at room temperature for 20 min, luminescence was measured with a SpectraMax M5 spectrofluorometer.

### The detection of cellular hepatitis C receptors on HLCs

HCV infection requires several types of cellular receptors to permit viral entry [34]. Cellular receptors essential for HCV infection such as Claudin-1, Occludin, SR-BI, CD81 were detected using quantitative RT-PCR and immunofluorescent technique. Mature HLCs were seeded on 24-well plate and maintained until 80 % confluence in Williams’ Media E, 10 % FBS. Cells were fixed and stained with fluorescent-conjugated antibodies raised against Claudin-1, Occludin, SR-BI or CD81.

### Production of HCV from JFH-1 (HCVcc)

Cell culture based hepatitis C virus (HCVcc genotype 2a) was prepared from JFH-1 system [35]. The JFH-1 plasmid was propagated in *E Coli* and extracted using NucleoBond Xtra Midi plasmid (MN, Germany). The plasmids were purified and linearized by a restriction enzyme XbaI, and used as a template for JFH-1 RNA synthesis. The full length HCV RNA was synthesized by TranscriptAid T7 High Yield Transcription Kit (Thermo Fisher Scientific, MA) following the manufacturer’s instruction. HCVcc was produced by transient transfection of JFH-1 RNA into Huh7 cell using either lipofectamine 3000 (Invitrogen, MA) or electroporation (Gene Pulser Xcell Electroporation Systems, Bio-Rad, CA). HCV in Huh7 supernatant was passed through 0.45 μm syringe filter, concentrated by sucrose gradient ultracentrifugation and stored at −80 °C for future use.

### The infection of HCVser and HCVcc to HLCs

HCV-positive sera were collected from patients with chronic HCV infection at Ramathibodi Hospital, Mahidol University. The collection of leftover blood specimen was approved by the Ethics Committee on Research Involving Human Subjects (2556/250). All subjects are adults and provided written informed consent. Serum from individual patient with HCV load > 10^6^ IU/mL was selected to infect HLCs. These sera carried HCV genotypes 1a, 1b, 3a, 3b, 6f and 6n that were prevalent in Thailand. For HCVser and HCVcc infection, HLCs and Huh7 cells were seeded on 6-well plate in Williams' Media E, 10 % FBS until 80 % confluence. Some host cells were pretreated with 1 μM or 5 μM α-tocopherol for 24 h prior to HCVser infection. For HCVser infection, 50 μL of infected serum in 2 mL of medium was added to host cells. For HCVcc infection, concentrated JFH-1 HCV load at 10^7^ IU/mL in 2 mL of medium was added to host cells. After 24 h incubation, the infected cells were washed 6–10 times with 0.1 % BSA in PBS and reconstituted with 2 mL complete growth medium.

### The long-term maintenance of HCV infection

For long-term HCV infection assay, HLCs and Huh7 were infected with HCVcc or HCVser. After 24 h incubation, the culture medium was renewed with 10 % FBS, DMEM/F12. The HCV-containing supernatant was collected ever 4–6 days and HCV RNA was detected using RT-PCR. During long-term cultivation process, HLCs and Huh7 cell were maintained in complete medium with 1 % concentrated lipids (Life Technologies, USA) and 1 μM (α-tocopherol). The HCV production was confirmed using the Abbott real-time HCV assay (Abbott Diagnostics, Illinois, USA).

### The detection of negative and positive stands of HCV RNA

HCV RNA in the supernatants was extracted with NucleoSpin RNA Virus isolation kit (MN, USA); while the intracellular RNA was extracted with illustra RNAspin Mini RNA Isolation Kit (GE Healthcare, Asia Pacific). The stand specific primers were designed using Vector NTI version 11.5 (Invitrogen, USA). The primers for positive strand are: 5′-CCCTGTGAGGAACTACTGTCTTCACGCA-3′ and 5′-CTCGCAAGCACCCTATCAGG-CAGTAC-3′. The primers for the negative strand are: 5′-GATGTACCCCATGAGGTCGG-3′ and 5′-GCGCGACAAG-GAAGACTTCG-3′ [36]. The viral RNA and total RNA from infected host cells were converted to cDNA with the ImProm-II reverse transcription system (Promega, WI). For HCV (-) stand, one microgram of isolated RNA was incubated with 12.5 μM HCV ‘reverse’ primers in a total volume of 5 μL at 70 °C for 5 min and chilled on ice-water immediately for at least 5 min. The reverse transcription mix (15 μL of 5× reaction buffer, 25 mM MgCl_2_, 2 mM dNTP Mix, 40 U/μL RNasin ribonuclease inhibitor, and 200 U/μL Improm-II™ RT) was added to the RNA-primer mix to make a total volume of 20 μL. The mixture was incubated at 25 °C for 5 min, and 42 °C for further 1 h. The RT reaction was terminated by heating at 70 °C for 15 min followed by chilling on ice. The HCV cDNA was amplified by KAPA SYBR® FAST qPCR Kits (Kapa Biosystems, UK) and 2.5 μM HCV ‘forward’ primer at 95 °C for 30 s, 66 °C for 45 s and 70 °C for 90 s. Hepatitis C virus (+) stand was quantified by real-time RT-PCR using the m2000sp and m2000rt instruments and RealTime HCV kit (Abbott Diagnostics, Illinois, USA).

### Statistical analysis

All results of experiments were performed in triplicated. Data were expressed as means ± SD. Data from quantitative real-time RT-PCR were evaluated for statistical significance using either students’ *t* test or ANOVA for difference followed by post-hoc tests (Tukey’s HSD) between the treatments and their respective controls. * and ** represented statistically different data with *p* < 0.05 and *p* < 0.01 respectively.

## Results

### Reprogramming human bone marrow mesenchymal stem cell to iPS cells

**Fig. 1:**
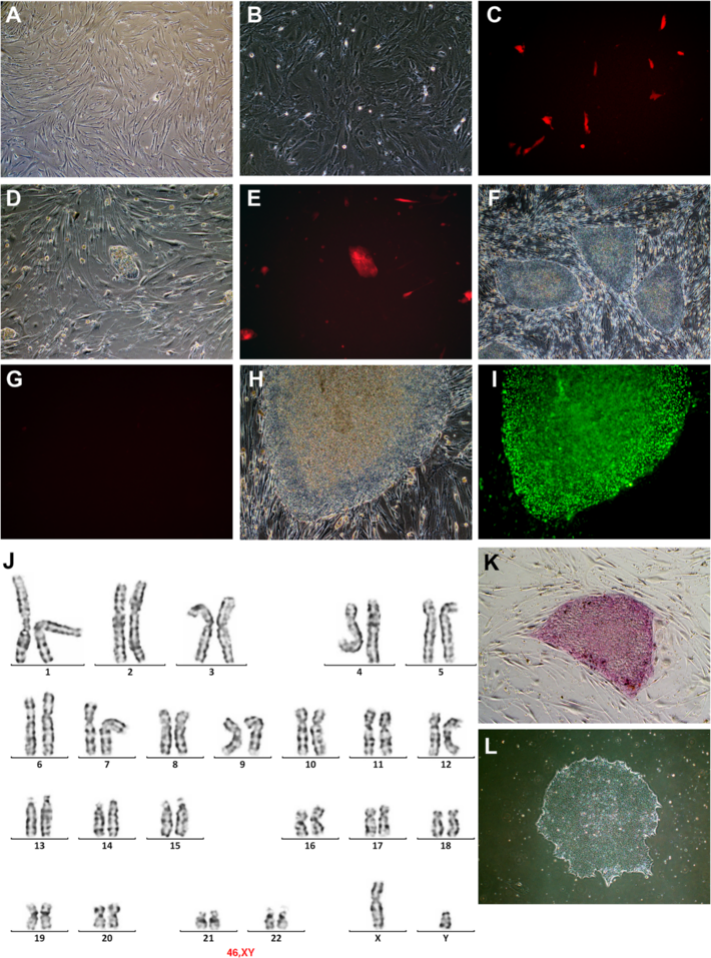
The generation of human iPS cell from hMSC. hMSC isolated from healthy donor at 2^nd^ passage had spindle-shape (**a**). hMSCs after transduced with polycistronic OSKM-dTomato lentivirus for 48 h was observed under a light microscope (**b**) and visualized for the staining of dTomato under a fluorescent microscope (**c**). Five days after the transduction, the transduced cells transformed to epithelial-like morphology forming clusters of dTomato-positive cells on day 7 (**d**, **e**). The cell colonies expanded and became tightly packed (**f**). The exogenous expression of OSKM were silenced and confirmed by negative dTomato (**g**). After achieving fully reprogramming, the stable colonies were observed (**h**) and expressed pluripotent surface marker TRA-1-60 (**i**). The iPS colonies were analyzed for karyotype (**j**). The colony was positive for alkaline phosphatase activity (**k**) and maintained in feeder-free condition (**l**)

hMSC isolated from aspirated bone marrow exhibited spindle-shaped morphology at 90–95 % confluence (Fig. 1a). After infecting hMSCs with polycistronic OSKM-dTomato lentivirus particles for 48 h, the transduced hMSCs expressing OCT4, SOX2, KLF4 and C-MYC were determined by positive dTomato (Fig. 1b and c). The dTomato-positive cells were detached and seeded on mitomycin c-treated MEF feeder at 2 × 10^5^ cells/10-cm dish. Cell morphology of dTomato positive population transformed from spindle-shape to epithelial morphology around day 5 post-transduction (Fig. 1d). These cell populations divided and formed a small cluster of dTomato after 2-3 d (Fig. 1e). The transduced colonies expanded and transformed to a tightly packed colony with a high nucleocytoplasmic ratio (Fig. 1f). Upon reaching full reprogramming, dTomato expression was silenced and reprogramming colonies were negative for dTomato on day 13 as monitored under a fluorescence microscope (Fig. 1g). The transduced cells reached iPS as determined by their positive pluripotent surface marker TRA-1-60 using a live staining technique (Fig. 1h and i). The TRA-1-60 positive colonies were selected and picked into a new MEF-seeded plates for expansion and characterizations.

### Identification of pluripotent markers in reprogramming cells and iPS cell characterizations

**Fig. 2:**
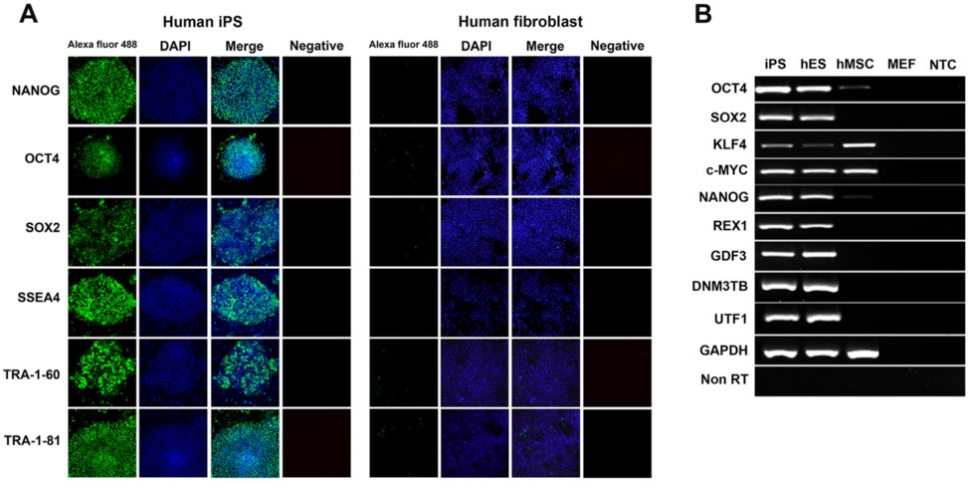
Identifications of pluripotent markers in human iPS cells derived from hMSCs. Newly established iPS cells were stained with monoclonal antibodies against pluripotent protein markers, e.g., Nanog, Oct-4, Sox2, SSEA4, Tra-1-60 and Tra-1-81. The iPS cells were counter stained with DAPI and visualized under a fluorescent microscope. Human fibroblast served as negative controls (**a**). To elucidate endogenous expressions of pluripotent markers in human iPS cells, specific primers were designed to detect the endogenous expression of Oct4, Sox2, Klf4, c-MYC, Nanog, REX1, GDF3, DNM3TB and UTF1 (**b**). The iPS cells (lane 1) expressed all pluripotent genes similarly to those of hES (BG01V: lane 2). Normal hMSC expressed only Klf4 and C-MYC (lane 3). PCR products from mouse embryonic fibroblast sample (lane 4) and NTC (lane 5) served as negative controls

TRA-1-60-positive colonies were passaged and maintained on MEF in ES medium until reprogramming cells achieved a typical human ESC morphology. The cell morphology consisted of a sharp border, flat, dense colony, high nucleocytoplasmic ratio and prominent nucleoli (Fig. 1h). The karyotype analysis confirmed no chromosomal alteration (Fig. 1j). The establishment of human iPS cell was confirmed using alkaline phosphatase (AP) activity, immunofluorescent staining of pluripotent markers and the expression of pluripotent genes (Fig. 1k). The reprogramming cells were maintained in E8 medium with feeder-free condition on Geltrex-coated dishes (Invitrogen, USA) prior to the analysis (Fig. 1l). The colony of reprogramming cells highly expressed essential pluripotent marker proteins, OCT4 and SOX2, required for maintaining pluripotent status. The expressions of OCT4 and SOX2 targeted proteins such as the self-renewal transcription factor NANOG, transmembrane proteins TRA-1-60 and TRA-1-81, and a cell surface glycoprotein SSEA4 were also expressed in the reprogramming cells. Human fibroblasts were used as negative controls for non-iPS cells (Fig. 2a). Exogenous OCT4 and SOX2 produced from the lentivirus were active at the early stage of cell reprogramming. To confirm the maintenance of endogenous OCT4 and SOX2 expression, specific OCT4 and SOX2 primers were designed to detect only the mRNA from endogenous source. The RT-PCR analysis confirmed the transduced cells carried endogenous OCT4 and SOX2 (Fig. 2b). Other pluripotent markers such as REX1, NANOG, GDF3, DNM3TB and UFT1 were expressed in the transduced cells and compared with those in human ES cell (BG01V) and non-transduced hMSCs (Fig. 2b). GAPDH and NTC served as positive and negative controls for PCR products.

### Spontaneous differentiation and teratoma formation

**Fig. 3:**
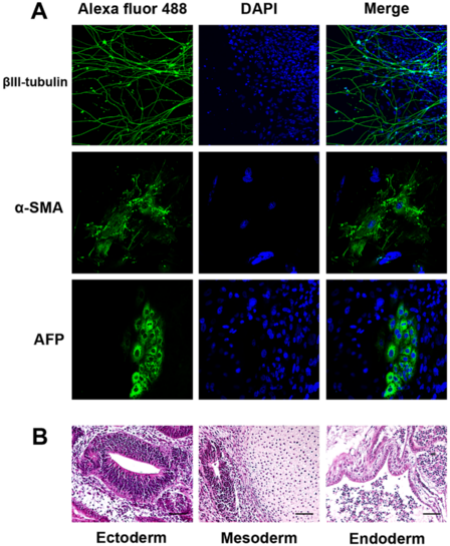
The pluripotency of human iPS cell derived from hMSCs. The iPS cells were cultured in complete medium with 20 % FBS or injected into nude mice to investigate in vitro and in vivo differentiation. The spontaneous differentiation of iPS cells appeared heterogeneous cell morphology. After staining with specific antibodies, differentiated cell positive for βIII-tubulin (ectoderm), α-smooth muscle actin (mesoderm) and α-fetoprotein (endoderm) (**a**). In vivo differentiation of iPS cell, histological examination of teratoma in nude mice after infected with iPS cells. The teratoma section contained various types of tissue including neural rosette (ectoderm), mesoderm-derived smooth muscle, adipose tissue, cartilage (mesoderm) and endoderm-derived gut-like epithelial (endoderm) (**b**)

To determine whether these newly established iPS cells could differentiate into all three germ layers, the iPS cells were evaluated for ex vivo and in vivo differentiation. For spontaneous ex vivo differentiation, iPS cells were maintained in suspension culture with embryoid bodies formation within a week. After 2 weeks, embryoid bodies were seeded on gelatin-coated culture plates to allow cell adhesion and migration. The adherent cells displayed various morphologies (Fig. 3a). Immunofluorescent staining revealed differentiated cell positive for βIII-tubulin (ectoderm marker), vimentin (mesoderm marker) and α-fetoprotein (AFP, endoderm marker) (Fig. 3a). These indicated the differentiation to several lineages. For teratoma formation, one million iPS cells were resuspended in DMEM 10 % FBS and injected subcutaneously into nude mice. Four weeks after injection, teratoma was formed at the injection site. Histological examinations revealed that the teratoma contained several tissues (Fig. 3b), e.g., neural rosette (ectoderm) (Fig. 3b, left panel), mesoderm derived smooth muscle, adipose tissue, cartilage (mesoderm) (Fig. 3b, middle) and endoderm-derived gut-like epithelial (endoderm) (Fig. 3b, right). Taken together, iPS cells derived from hMSCs not only support the spontaneous ex vivo differentiation but they can form teratoma in vivo.

### The hepatocyte-like cell derived from iPS cell expressed hepatic phenotypes

**Fig. 4:**
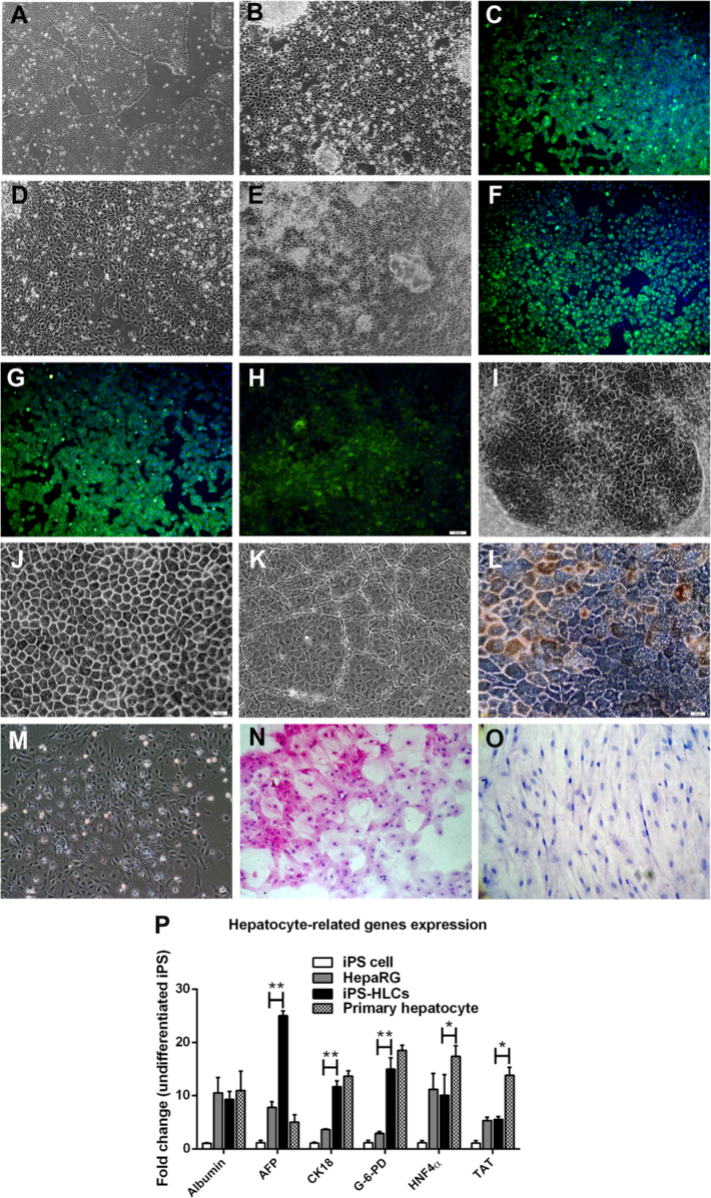
The direct differentiation of iPS cell into fully the functional homogenous HLCs. The iPS cells in feeder-free condition at 60 % confluence (**a**) were differentiated into definitive endoderm with round-shaped morphology (**b**). During differentiation induction, cells were positive for Sox17 (**c**). During anterior definitive endoderm (ADE) development, the differentiated cells underwent epithelial to mesenchymal transition (EMT) (**d**). The ADE cells with oval-shape (**e**) served as hepatic progenitors. Differentiated cells were positive for α-fetoprotein (**f**), albumin (**g**) and HNF-4α (**h**). The homogenously mature HLCs exhibited polygonal morphology (**i**), cord-like structure with bile canaliculi (**j**) and hepatic sinusoidal-like structures (**k**). The HLCs were positive for bile pigment (**l**). The 2^nd^ passage of HLCs still maintained the hepatic morphology (**m**). HLCs and hMSCs were examined for glycogen storage using PAS assay (**n**, **o**). HLCs, HepaRG and primary human hepatocyte were compared for the expressions of hepatocyte selective genes (**p**). Data were presented as a fold changes over the undifferentiated iPS cells. * and ** represented statistical different data with a *p* value <0.05 or <0.01 respectively

Human iPS cells were cultured in feeder free condition until reaching 70 % confluence before hepatic induction (Fig. 4a). During hepatic differentiation, iPS cells were monitored daily for morphological changes. Initially, iPS cells were differentiated into definitive endoderm (DE) cells (Fig. 4b) that shared the same precursor with all endodermal organs (pancreas, liver, lung, thyroid and gut) [37]. The differentiated cell transformed into round-shaped morphology (Fig. 4b) with positive staining for SOX17, an endoderm differentiation marker (Fig. 4c). The second stage, definitive endoderm cells were developed to anterior definitive endoderm (ADE) with cell morphology underwent epithelial to mesenchymal transition (EMT) [38]. Cells displayed the mesenchymal-like morphology (Fig. 4d). At the third stage of differentiation, ADE cells became hepatic progenitors (Fig. 4e). The differentiated cell at this stage was confirmed with the expression of alpha-fetoprotein (AFP) (Fig. 4f), a characteristic of hepatoblast [31]. At maturation, hepatoblasts developed into functional hepatocyte-like cells (HLCs). HLCs shared both fetal and adult hepatocyte markers, e.g., albumin and minimal level of alpha-fetoprotein (Fig. 4g). The expression of HNF4-α, found only at the end of maturation, revealed that the cells fully developed into mature hepatocytes (Fig. 4h). After 4-week differentiation, human iPS cell developed into a near homogenous population with more than 95 % pure hepatocyte-like cell. HLCs exhibited typical hepatocyte morphology, e.g., a polygonal shape, a dark cytoplasm with large nuclei containing nucleoli and bi-nucleated (Fig. 4i). Mature HLCs maintained in Williams' Media E exhibited homogeneous population (Fig. 4j), hepatic triad-like structure (Fig. 4k) and some populations produced bile pigment (Fig. 4l). HLCs exhibited polygonal morphology during passages (Fig. 4m). HLCs showed the capability to synthesize and store glycogen greater than did hMSCs as detected by Periodic acid Schiff staining (Fig. 4n and o). The HLCs were also positive for gene expressions of α-fetoprotein, albumin and HNF4-α using quantitative RT-PCR (Fig. 4p). For major metabolic functions in hepatocyte involving carbohydrate, lipid and amino acid metabolism, HLCs expressed both glucose-6-phosphate dehydrogenase (G-6-PD) and tyrosine aminotransferase (TAT). The expression levels of basic hepatocyte markers (i.e., ALB, AFP, HNF4-α, and TAT) were increased between 10–20 folds over those from undifferentiated iPS cells.

### The elevated expression levels of most basal CYP450 isozymes in mature HLCs were inducible with prototypic inducers

**Fig. 5:**
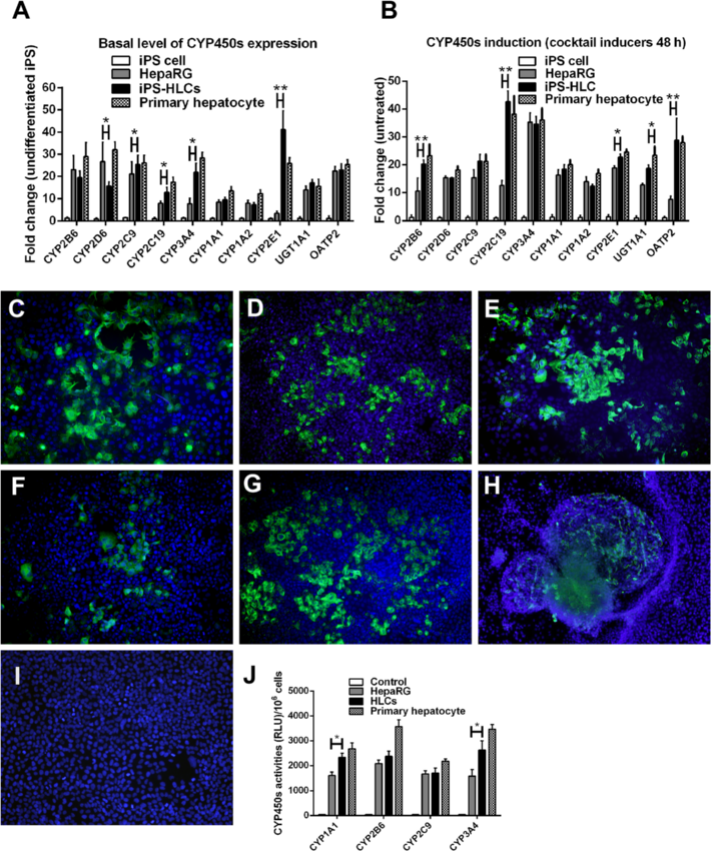
The detection of function CYP450 isoenzymes in HLCs. Mature HLCs were investigated for CYP450 expression, protein levels and activities. HLC, HepaRG and primary hepatocyte were studied for the expression of CYP450 isoenzymes and presented as fold changes over the undifferentiated iPS cells (**a**). The CYP450 induction with the cocktail of inducers was evaluated and presented as fold changes over the corresponding untreated cells (**b**). * and ** represented statistical different data with a *p* value <0.05 or <0.01 respectively. The levels of CYP3A4 (**c**), CYP2B6 (**d**), CYP1A1 (**e**), CYP2C9 (**f**) and CYP2E1 (**g**) in untreated HLCs and inducer-treated HLCs were measured. Bile canaliculi marker (MRP2) was observed in HLCs (**h**), but not in human fibroblasts (**i**). The CYP450 activities (**j**) were assayed and presented as a relative light unit (RLU) over the corresponding untreated cells

To achieve fully functional hepatocyte, mature HLCs were maintained in 10 % FBS Williams’ Media E for 3 d and the cells were screened for CYP expression. HLCs expressed a number of CYP isozymes as illustrated by immunostaining and quantitative real-time PCR. The basal mRNA levels of CYP2B6, CYP2D6, and CYP2C9 in HLCs were 22-, 25-, and 20-fold that of HepaRG cells respectively; while CYP3A4, CYP2C19, CYP1A2 were around 10 folds. CYP2E1 in HLCs was up-regulated to 40-fold that of the undifferentiated iPS cells (Fig. 5a). Other isozymes achieved comparable expression levels to those of HepaRG and the primary human hepatocytes. To evaluate the inducibility of these CYP isozymes, the HLCs were incubated with the cocktail of chemical inducers (20 μM rifampicin, 50 μM omeprazole, 1 mM phenobarbital and 88 μM ethanol). The expressions of CYP2B6, CYP2C9, CYP2C19 and CYP3A4 in HLCs were increased to 2, 2, 4, 3.5 folds respectively after the induction with the cocktail. In comparison, the expressions of CYP2B6, CYP2C9 and CYP3A4 in HepaRG were increased to 2, 2, 3.5 folds and comparable to those in HLCs (Fig. 5b). In particular, some CYP isozymes, e.g. CYP2B6, CYP2C9, CYP2C19 and CYP2E1 were more inducible in HLCs than in HepaRG cells. The expression levels of CYPs in primary human hepatocytes were dramatically decreased post-induction by 72 h. In addition to CYP expressions, HLCs contained high levels of organic anion transporting polypeptide 2 (OATP2). The elevated OATP2 expression could be extensively up regulated to 3 folds. For protein determination, more than 50 % of HLC populations were positive for intracellular CYP1A1, CYP3A4, CYP2B6 and CYP2E1 proteins (Fig. 5c–g). The immunofluorescent straining demonstrated the increasing expression of major CYP proteins (Fig. 5c–g), e.g., CYP3A4, CYP2B6, CYP1A1, CYP2C9 and CYP2E1. HLCs expressed multidrug resistance-associated protein 2 (MRP2) that represented the canalicular (apical) membrane of the hepatocyte as a biliary transport (Fig. 5h). The hMSC served as a control of non-hepatic cell (Fig. 5i). The increasing of CYP450 activities was investigated in hepatocytes (Fig. 5j). Mature HLCs population could proliferate, but still maintained hepatic morphology without losing basal levels of hepatic related gene expression.

### HLCs expressed all major hepatitis C virus cell associated receptors

**Fig. 6:**
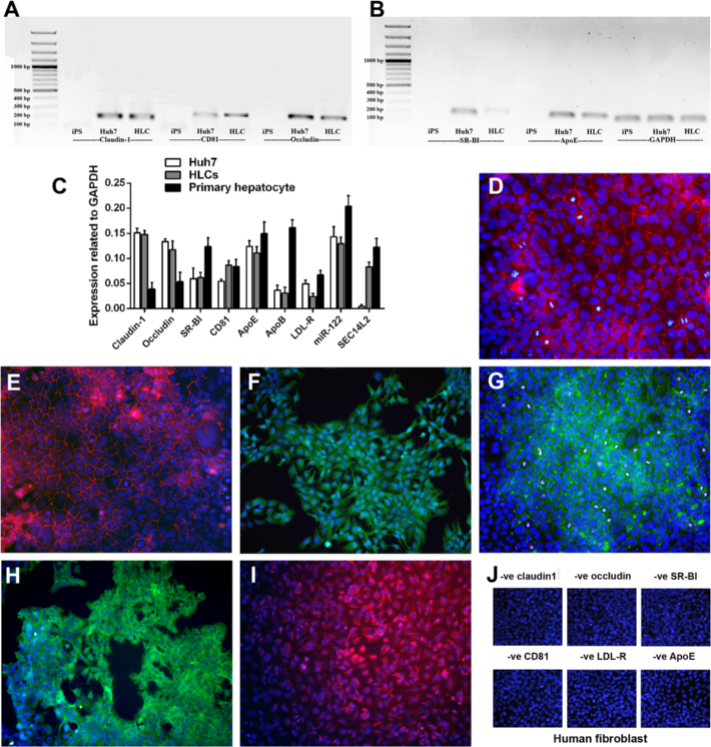
Identifications of HCV cell-associated receptors and essential host factors on HLCs. Gel electrophoresis from RT-PCR products confirmed the expression of major HCV cell-associated receptors in iPS cell, Huh7 and HLCs (**a**, **b**). The expression levels of HCV cell-associated receptors and essential host factors (claudin-1, occludin, SR-BI, CD81, ApoE, ApoB, LDL-R, miR-122 and SEC14L2) in HLCs, HepaRG and primary hepatocyte were determined using real-time RT-PCR (**c**). * and ** represented statistical different data with a *p* value <0.05 or <0.01 respectively. Cellular localization of HCV cell-associated receptors was visualized using immunofluorescent straining. HLCs were visualized for claudin-1 (**d**), occludin (**e**), SR-BI (**f**), CD81 (**g**), LDL-R (**h**) and ApoE (**i**) in comparison to those observed in human fibroblasts (**j**)

A potential application for HLCs is for the study of pathogen-host cell interactions. The HLCs derived from iPS cells were evaluated for the capability to host HCV replication. The expression of HCV receptors is essential for virus hepatitis C viral entry. Mature HLCs expressed all major receptors for HCV entry (Fig. 6a–c), e.g., specific tight junctions: claudin-1, occludin; cell surface receptor: TAPA-1 (CD81); scavenger receptor B1 (SR-BI), ApoB and ApoE. The expression of specific HCV receptors was increased together with the maturation stage of HLCs. On the cell surface of cultured HLCs, the protein products of these receptors were also detected (Fig. 6d–i), e.g., claudin-1, occludin, SR-BI, CD81, LDL-R and ApoE. Human fibroblast served as a negative control (Fig. 6j).

### Hepatocyte-like cells support HCVcc virus production through JFH-1 system

**Fig. 7:**
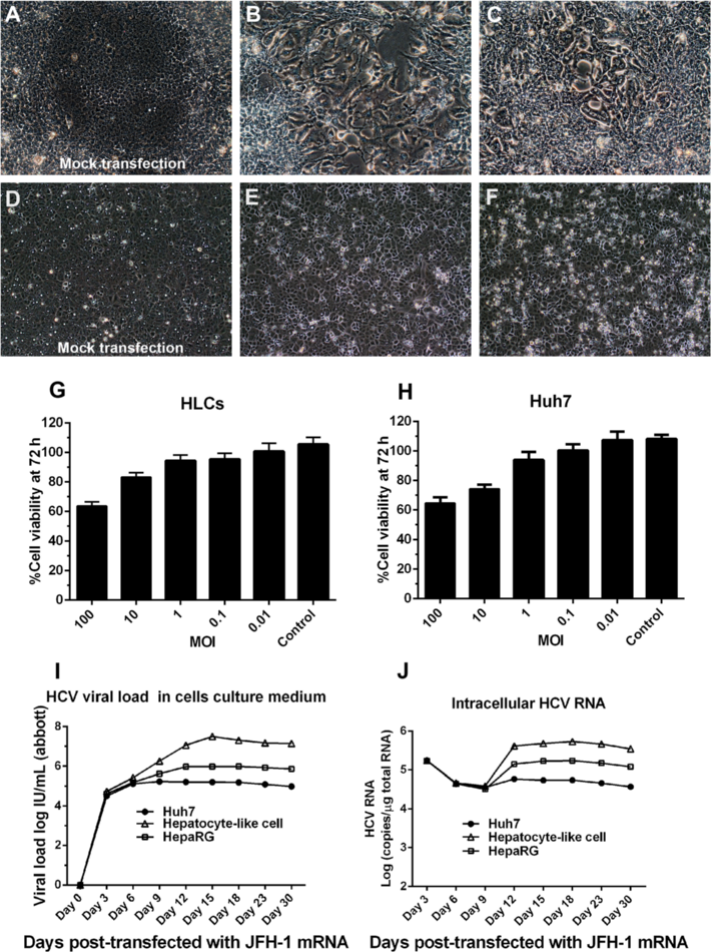
Production of HCVcc (JFH-1) in HLCs and the viral propagation kinetics. HLCs were either mock transfected (**a**) or transfected with JFH-1 RNA to produce HCVcc had induced cytopathic effect, cell aberrations (CPE) on day 7 (**b**) and syncytial formation (**c**) on day 14. In comparison, Huh7 cells were either mock transfected (**d**) or transfected with JFH-1 RNA on day 7 (**e**) and 14 (**f**). Cell viability assay using MTT in HLCs (**g**) and Huh7 (**h**) corresponded to the MOI of HCVcc. The kinetics of HCV production in the culture medium were investigated using Abbott real-time HCV assay (**i**); while the kinetics of HCV replication were investigated using stand-specific real-time RT-PCR (**j**) in Huh7, HLCs and HepaRG cells

To determine if HLCs could replace the Huh7 cells to host HCV, HLCs were transfected with JFH-1 RNA using liposomal transfection [14]. Seven days after transfection, we found cytopathic effect with the aberration of cytoplasm in HLCs, but not with mock transfection (Fig. 7a and b). These cells had lower growth rate, aggregated and formed clusters of syncytial cells (Fig 7c). These findings suggested that HCV might re-infect the HLCs in high titer. We could not observe the CPE nor the aberration of cytoplasm in mock transfection or JFH-1 transfected Huh7 cell on day 7 and day 14 (Fig. 7d–f). Conditioned medium from HLCs and Huh7 was harvested every 3 d and filtered through a 0.45 μM syringe filter. The suitable multiplicity of infection (MOI) was optimized using 10-fold HCV dilution. Cell viability was determined with MTT assay (Fig. 7g and h). The HCV viral load was determined and compared between different cell lines (Fig. 7i). The kinetics of viral production varied depending on the hosting cells. The maximal HCV production was achieved between 12-15 d post-transfection. HCV viral load from Huh7 was about 10^5^ IU/mL but HLCs could provide up to 10^7^ IU/mL (Fig. 7i), comparable to those offered (10^8^ IU/mL) by the transfected Huh7.5 [17]. The detection of HCV in HLCs supernatant was maintained beyond 1 month. In transfected Huh7 cells, HCV viral load was decreased to 10^4^ IU/mL within 2 weeks and completely vanished in two months (Fig. 7i and j).

### Completion of the wild-type HCV life cycle in HLC and the production of HCV progeny

**Fig. 8:**
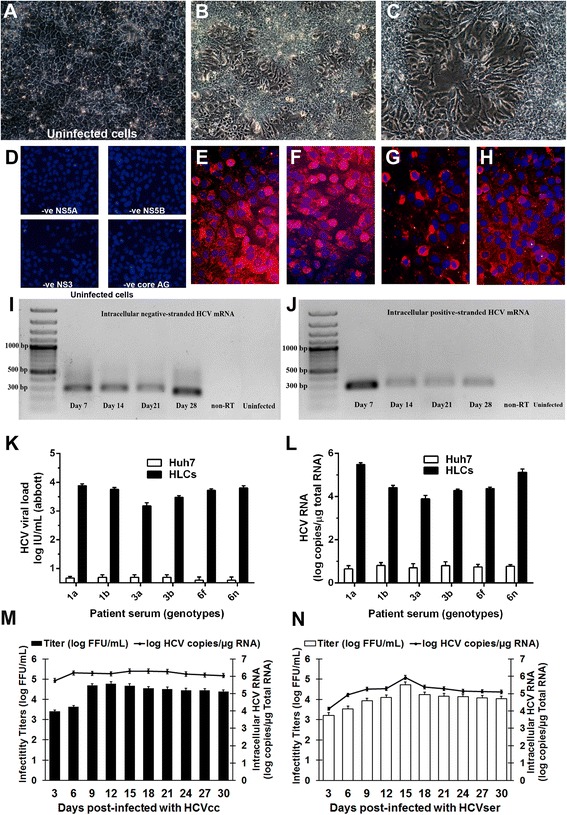
HLCs supported entire HCVcc and HCVser life cycle. Uninfected HLC (**a**) or HLCs infected with HCVcc (JFH-1) or HCVser (genotype 1a) were observed for CPE and cytoplasmic aberrations (**b**, **c**). Uninfected HLCs (**d**), or HLCs after the infection for 14 d were investigated for HCV non-structural proteins, e.g., zinc-binding phosphoprotein (NS5A) (**e**), viral RNA polymerase (NS5B) (**f**), viral protease enzyme (NS3) (**g**) and the HCV structural protein (core antigen) (**h**). The intracellular negative-strand RNA represented the HCV replication (**i**). The intracellular positive-strand RNA level represented HCV entry and production (**j**). HCVser viral load (**k**) and intracellular RNA (**l**) in Huh7 and HLCs after natural HCV infection were monitored. The infectivity titers of HCVcc (**m**) and HCVser (**n**) were examine using fluorescent focus forming units (FFU/mL); while the intracellular HCV RNA was determined by quantitative real-time PCR (RNA log copies/μg of total RNA)

The naïve HLCs and naïve Huh7 cells were infected with either HCVcc (JFH-1 genotype 2a) or with HCVser of various genotypes including 1a, 1b, 3a, 3b, 6f and 6n. HCVcc could be obtained from the earlier transfection of HLCs or Huh7 with JFH-1 RNA. HCVser was collected from chronic hepatitis C patient. The cytopathic effect (CPE) in the infected cells was observed on both day 7 and 14 after infection (Fig. 8a–c). The cytoplasmic localization of non-structural proteins: NS3A, NS5A, NS5B and structural protein: HCV core antigen was clearly detected. Therefore, HLCs allowed both HCV entry and the transcription of viral RNA (Fig 8d–h). To ensure that the HLCs support entire HCV life cycle, the total RNA prepared from infected HLCs and Huh7 cell was investigated for the presence of HCV negative-strand RNA using specific RT-PCR primers. The viral RNA was maintained in infected cells up to 4 weeks in HLCs 2 weeks in Huh7 cells. The presence of positive-strand of viral RNA was measured in the supernatant from the infected cells using RT-PCR analysis (Fig. 8i and j). The HCVcc viral loads were 10^7^ IU/mL and 10^4^ IU/mL from the supernatants of HLCs and Huh7, respectively. The levels of intracellular HCV negative–strand RNA and positive-strand RNA were determined by reverse transcription PCR every week after transfection until 4 weeks. The negative-strand RNA was merely detectable on day 7 and increased on day 14 (Fig. 8i and j). For HCVser, HLCs allowed viral propagation as determined by the increasing HCV particles in culture medium (Fig. 8k) and the detection intracellular HCV RNA (Fig. 8l). Huh7 cell, in contrast, could barely hosted HCV derived from patient serum. The HCVser viral loads were 10^4^ IU/mL from the supernatants of HLCs. The kinetics of viral replication post-infection with either HCVcc (JFH-1) or HCVser (genotype 1a) in HLCs were determined using HCV titer and intracellular HCV RNA. The kinetics of HCV production suggested that HLCs supported complete HCV replication of both HCVcc and HCVser (Fig. 8m and n). However, HLCs did allow HCVser replication beyond 4 weeks (Fig. 8n). The immunostaining and RT-PCR analysis confirmed that the entire replication cycle and formation of HCV progeny took place in HLC and the supernatants from the infected cultures could efficiently infect naïve HLCs and Huh7 cells.

### The α-tocopherol-pretreated HLCs improved HCV viral load

**Fig. 9:**
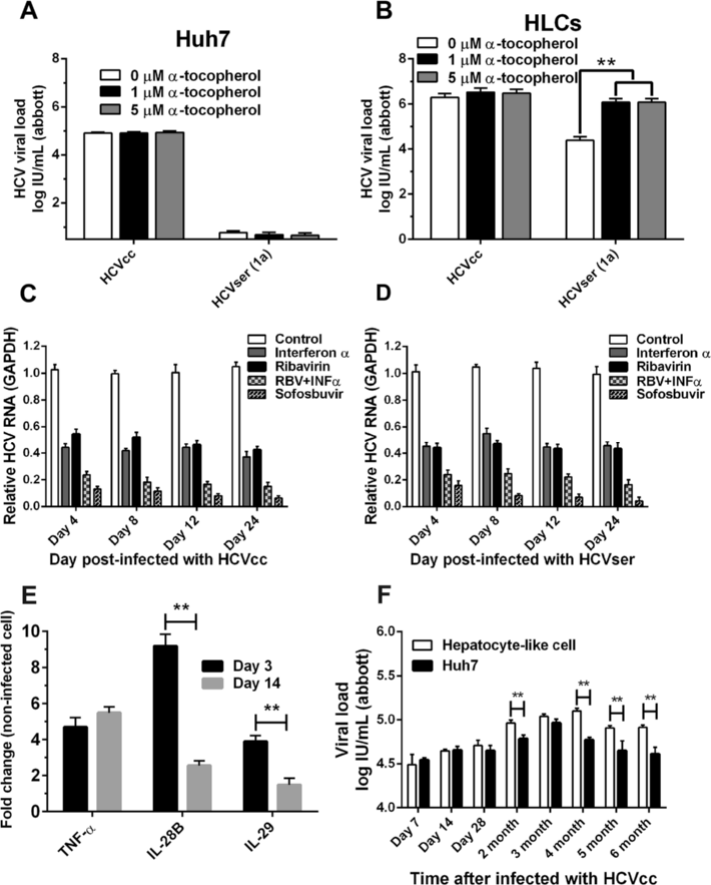
The enhancing effect of α-tocopherol and the inhibitory effects of anti-HCV drugs on HCV replication. Huh7 (**a**) and HLCs (**b**) were treated with 0, 1, 5 μM α-tocopherol 24 h prior to infection with HCVser and assayed for HCV viral load. For anti-viral treatment, HLCs were either infected with HCVcc or HCVser (*n* = 3) for 24 h prior to the treatment with ribavirin, Interferon-α or sofosbuvir for 7 d. Copies of HCV RNA in lysates were quantified by real-time PCR (**c**, **d**). Infected HLCs were assayed for inflammatory markers (**e**). For long-term maintenance of HCVcc in HLCs, the supernatants of the infected HLCs were measured for viral load up to 6 months (**f**)

The effect of α-tocopherol to HCVser infection was investigated using HLCs and Huh7 cell line. After the pretreatment with 1 μM or 5 μM α-tocopherol for 24 h and infection with either HCVcc (JFH-1) or HCVser genotype 1a, the infected HLCs and Huh7 cells were further maintained for 14 days. The HCVser viral load was raised to 10^6^ IU/mL in HLCs but the HCVcc viral load was not affected by α-tocopherol. In Huh7 cell, α-tocopherol did not improve the HCV viral load in HCVser infection and HCVcc (JFH-1) infection (Fig. 9a and b).

### The efficacy study of anti-HCV drugs in infected HLCs

To screen for the sensitivity of infected HLCs to anti-HCV agents, naïve HLCs were infected with 10^6^ IU/mL of either HCVcc (from conditioned medium of HLCs) or HCVser. Different anti-HCV agents or their combinations, e.g., ribavirin (20 μM), IFN-α (10 IU/mL), and sofosbuvir (PSI-7977, 200 nM) were incubated with the 24-h infected cells for 7 d. The HCV RNA expression in HCVcc and HCVser served as drug response. The intracellular HCV RNA was determined by real-time qPCR at 4, 8, 12, and 24 d after the infection initiation. Ribavirin, IFN-α and sofosbuvir were not cytotoxic up to 100 μM, 10 IU/mL and 100 μM respectively. Either ribavirin or INF-α decreased HCV RNA to 50 % in Huh7 and HLCs. The combination of INF-α and ribavirin decreased viral RNA to 20 %. Sofosbuvir decreased viral RNA below 20 %. (Fig. 9c and d). The IC_50_ of INF-α toward HCV RNA level in HLCs and Huh7 cells were 5.3 and 2.4 IU/mL respectively (*p* < 0.01). The IC_50_ of ribavirin in HLCs and Huh7 were 6.9 and 3.2 μg/mL respectively (*p* < 0.01). The IC_50_ of sofosbuvir in HLCs and Huh7 were 96 and 92 nM respectively (*p* < 0.01) (data not shown).

### Host inflammatory response and the maintenance of long-term HCVcc propagation in HLCs

Relative real-time qPCR on days 3 and 14 revealed that the expression of inflammatory markers, e.g. TNF-α, IL-28B and IL-29 were inducible during the infection. Particularly, IL-28B expression in response to HCV infection was observed earlier only in primary human hepatocyte, but also in our HLCs model (Fig. 9e). Long-term HCVcc production was evaluated by infecting naïve HLCs with viral load 10^7^ IU/mL for 72 h. The cell culture medium that had been replaced every week still carried HCV particles up to 6 months. The kinetics of HCV represented the new viral particles releasing from infected cell to the medium (Fig. 9f). At 4-week post-infection, the HCVcc viral load varied from 10^4^ to 10^5^ IU/mL and increased at 2-3 months later to 10^5^ IU/mL. The HCV viral production was still maintained at 10^4^-10^5^ IU/mL up to 5 months. Until 6 months, most of the HLCs died in accordance with the viral CPE.

## Discussion

Our study demonstrated that HLCs could host HCV for at least 6 months while retaining the capability to allow a complete viral life cycle. In these regards, HLCs are superior to the classical HCV host, the Huh7 cells. Despite HLCs had been studied in association with HCV, only a brief infection with JFH-1 had been addressed. These earlier studies employed immature and heterogeneous HLCs derived from iPS/ES cells as host cells for HCVcc infection [27, 28, 30]. These cells did not express all of major functional CYP450s and were functionally immature that constrained the study of HCV-host interactions [39–42]. We expanded this observation by elucidating the mature HLCs not only as a viral target, but as a producer of new viral particles to complete a viral life cycle.

We modified the previous hepatic differentiation protocol [31, 43, 44] to achieve homogenous population of fully functional HLCs by extending the maturation step. The differentiated cells were challenged with a cocktail of CYP inducers. During hepatic differentiation, the differentiated cells expressed hepatic lineage markers (Sox17, AFP and albumin). In final step, we observed the homogeneous of hepatocyte-like cell with bile canaliculi, hepatic triad-like structure and bile pigments. HLCs were positive for hepatocyte selective markers, e.g., HNF-4α and tyrosine aminotransferase (TAT). The expression level of hepatic markers was higher than those of HepaRG cell, but were comparable to those of primary hepatocyte.

The xenobiotic biotransformation is a crucial and specialized function of hepatocyte. Previous studies could drive the differentiation of human iPS cells to only immature hepatocytes [45, 46]. Our study demonstrated that HLCs express phase I (CYP2B6, CYP2D6, CYP2C9 CYP2C19, CYP1A2 and CYP2E1), phase II drug-metabolizing enzyme (OATP2) and phase III drug transporters (MRP2) at levels comparable to those of primary human hepatocytes, but higher than HepaRG, the hepatocellular carcinoma cell line frequently used in pharmaceutical industry. The expression of all major CYP450s was inducible with the standard cocktail of inducers [33].

Cellular polarity is crucial for maintaining hepatic phenotype and the susceptibility to HCV [47]. At the end of hepatic induction, four types of HCV receptors on the basolateral membrane were detected, i.e., claudin-1, occludin, SR-BI and CD81. HCV particles triggered the increase in CD81 and claudin-1 endocytosis via clathrin [48]. Clathrin-dependent endocytosis and LDL metabolism involved in the processes of HCV entry [49–51]. ApoB and ApoE were involved in the production of HCVcc in Huh7 cells [52]. Both apolipoproteins and LDL-receptors on apical membrane correlated with HCV entry and emission from host cells [53]. The production of infectious particles depended on VLDL secretion pathway in hepatocytes [54]. We observed the increasing expressions of ApoE, ApoB and LDL receptors after maturation.

Since the discovery of JFH-1 replication in Huh7 cells [11], subclones of Huh7 (Huh7.5 and Huh7.5.1) had been developed as a replacement for primary hepatocytes. HCV has been classified into seven major genotypes and numerous of subtypes [55–57]. Nonetheless, JFH-1 is a single unusual HCV strain of genotype 2a. Other genotypes could not be maintained in Huh7 cells due to cell tropism and the restrict host range. The robust propagation systems of full-length HCV clones of the TN strain (1a) [58], H77 strain (genotype 1a) [59], JFH-2 (2a) and s310 strain (3a) [60] were developed but the construction of other HCV genotypes has not been success. HepG2, Hep3B and HEK293T cells allowed HCVcc replication [61–63]. However, these cells require exogenous host factors (e.g., miR-122, claudin-1 and ApoE) for complete propagation of HCVcc [63]. Novel culture models that can host various HCV genotypes are required. Our study showed that HCV production was sustained for six months in HLCs that had been either transfected with full-length JFH-1 RNA or infected with HCV genotype 1a, 1b, 3a, 3b ,6f and 6n. No requirement for exogenous expression of cellular host factors was required for the production of HCVcc. HCVcc and HCVser produced from HLCs could infect naïve hepatocytes.

The essential HCV host factors, miR-122 and SEC14L2, facilitated the propagation of HCV in cultured hepatocyte. SEC14-like protein 2 (SEC14L2) promoted HCVser infection by upregulating vitamin E-mediated protection against lipid peroxidation. SEC14L2 together with α-tocopherol transfer protein and cellular retinol-binding protein involved in cholesterol biosynthetic pathway [64]. Both miR-122 and SEC14L2 were highly expressed in HLCs and primary human hepatocytes, but not Huh7 cells, correlated with the capability to host HCVser propagation.

After transfection with HCV RNA or infection with patient serum, we found the CPE in both transfected and infected HLCs within 7-d post-transfection. HCV viral load in culture was 10^4^ IU/mL on day 3 and 10^8^ IU/mL on day 15; while the intracellular HCV RNA was detectable starting on day 3. Taken together, HLCs supported complete HCV life cycle from JFH-1 system and from patient serum.

Host cell immune response play a major role in antiviral activity which is naturally occurred in animal models but defective in current cell culture model of HCV [65]. HLCs were investigated for cellular immune responses by infecting with HCVcc for 14 d and inflammatory cytokines were detected on day 3 and day 14. The cellular response to HCV infection was clearly observed in HLCs. In HLCs, the expressions of inflammatory cytokines, e.g., TNF-α, IL-28B and IL-29 were up-regulated during the infection that mimicked the natural infection in the primary hepatocytes. IL-28B is associated with the sensitivity of IFN treatment of chronic hepatitis C patients [66, 67]. These results are typical of an ongoing inflammatory response in cells that could serve as indicators for subsequent antiviral strategies.

Our study implicated that our HLCs could substitute the primary hepatocytes to serve as a generalized host for HCV. A complete life cycle of the wild-type HCV could be driven in our culture system. With this encouraging observation, an array of applications of HLCs for the study of hepatocyte-pathogen interactions would be the next direction. The list of potential pathogens included hepatitis B and dengue viruses that have been conventionally limited to human biopsies, surgical resection, organ donation and hepatocellular carcinoma cells from clinical specimens. The obtained host-pathogen interactions would provide a platform for the screening of efficacious antiviral regimens in accordance with personalized medicine.

## Conclusions

Here, we have developed the functional homogenous HLCs starting from hMSCs through iPS cell reprogramming. These HLCs were confirmed for eliciting major hepatic functions including CYP450 activities. They exhibited the capability for hosting long-term HCVser infection in cell culture condition, bypassing the earlier limitation to the genotype 2a. Together with the expression of SEC14L2 and the addition of α-tocopherol, the HCVser infection in HLCs were further intensified. We demonstrated that the production of HCV genotype 1a, 1b, 3a, 3b ,6f and 6n were sustained for at least six months in these HLCs. The HCV-infection was controlled by the upregulation of TNF-α, IL-28B and IL-29. Taken together, this novel HCV culture could serve as a model for HCV biology, host immune response, and antiviral strategy.

### Ethics approval and consent to participate

Human mesenchymal stem cells (hMSC) were isolated from aspirated bone marrow of consenting healthy donors (*n* = 3). This procedure received an approval from the Ethics Committee on Research Involving Human Subjects at Ramathibodi Hospital, Mahidol University (2554/404). All subjects are adults and provided written informed consent.

## Additional file

Additional file 1: Primer sets and conditions used in quantitative real-time PCR (qPCR). (DOCX 15 kb)

## Abbreviations

HCV: Hepatitis C virus
JFH1: Japanese Fulminant Hepatitis 1
HCVcc: Hepatitis C Virus derived from cell culture
HCVser: Hepatitis C Virus derived from patient serum
HLCs: Hepatocyte-Like Cells
hMSC: Human Mesenchymal Stem Cell
iPSC: Induced Pluripotent Stem Cell
PCR: Polymerase Chain Reaction
CYP450: Cytochrome P450
INF: Interferon
RLU: Relative Light Unit
