# Supplementary material for: A robust model of natural hepatitis C infection using hepatocyte-like cells derived from human induced pluripotent stem cells as a long-term host
Source: Virol J. 2016 Apr 5;13:59. doi: 10.1186/s12985-016-0519-1 (PMC4820862; doi:10.1186/s12985-016-0519-1)
Supplement: Additional file 1: — Primer sets and conditions used in quantitative real-time PCR (qPCR). (DOCX 15 kb) [file 12985_2016_519_MOESM1_ESM.docx]

**Additional file 1. Primer sets and conditions used in quantitative real-time PCR (qPCR)**

| **Gene** | **Genbank**  **Accession** | **Sense primer**  **5’-----> 3’ (Tm °C)** | **Antisense primer**  **3’-----> 5’ (Tm °C)** | **Amplicon size (bp)** | **Annealing temp. (°C)** | **Putative function** |
| --- | --- | --- | --- | --- | --- | --- |
| **ALB** | NM_000477 | TGAGAAAACGCCAGTAAGTGAC (56.5) | TGCGAAATCATCCATAACAGC (54.7) | 265 | 60 | albumin |
| **AFP** | NM_001134 | GCTTGGTGGTGGATGAAACA (57.2) | TCCTCTGTTATTTGTGGCTTTTG (54.6) | 157 | 60 | α-fetoprotein |
| **CK18** | X12881 | GAGATCGAGGCTCTCAAGGA (57.9) | CAAGCTGGCCTTCAGATTTC (55.8) | 357 | 60 | cytokeration 18 |
| **G6PD** | U01120 | GCTGGAGTCCTGTCAGGCATTGC (58.1) | TAGAGCTGAGGCGGAATGGGAG (63.1) | 349 | 60 | glucose-6-phosphate dehydrogenase |
| **HNF-4α** | AY680696 | GCCTACCTCAAAGCCATCAT (56.4) | GACCCTCCCAGCAGCATCTC (62.9) | 256 | 60 | hepatocyte nuclear factor 4α |
| **TAT** | NM_000353 | TGAGCAGTCTGTCCACTGCCT (62.3) | ATGTGAATGAGGAGGATCTGAG (54.9) | 338 | 60 | tyrosine aminotransferase |
| **CYP2B6** | NM_000767 | ATGGGGCACTGAAAAAGACTGA (58.0) | AGAGGCGGGGACACTGAATGAC (63.5) | 283 | 60 | CYP2B6 |
| **CYP2D6** | NM_000106 | CTAAGGGAACGACACTCATCAC (56.6) | GTCACCAGGAAAGCAAAGACAC (58.1) | 289 | 60 | CYP2D6 |
| **CYP2C9** | NM_000771 | CCTCTGGGGCATTATCCATC (57.1) | ATATTTGCACAGTGAAACATAGGA (52.9) | 137 | 60 | CYP2C9 |
| **CYP2C19** | NM_000769 | TTCATGCCTTTCTCAGCAGG (56.8) | ACAGATAGTGAAATTTGGAC (47.9) | 277 | 60 | CYP2C19 |
| **CYP3A4** | AK298451 | GCCTGGTGCTCCTCTATCTA (57.6) | GGCTGTTGACCATCATAAAAGC (56.0) | 187 | 60 | CYP3A4 |
| **CYP1A2** | AF182274 | ACCCCAGCTGCCCTACTTG (61.8) | GCGTTGTGTCCCTTGTTGT (57.4) | 101 | 60 | CYP1A2 |
| **CYP2E1** | NM_000773 | ACCTGCCCCATGAAGCAACC (62.8) | GAAACAACTCCATGCGAGCC (58.9) | 246 | 60 | CYP2E1 |
| **UGT1A1** | BC128414 | GGAGCAAAAGGCGCCATGGC (65.6) | GTCCCCTCTGCTGCAGCTGC (66.6) | 178 | 60 | uridine diphosphate glucuronyltransferase 1A1 |
| **OATP2** | AJ132573 | GCCCACGCGTCCGACT (63.8) | ACAGAGCTGCCAAGAACATCT (57.6) | 277 | 60 | organic anion transporting polypeptide 2 |
| **Claudin-1** | NM_021101 | GTGGAGGATTTACTCCTATGCCG (59.1) | ATCAAGGCACGGGTTGCTT (59.1) | 165 | 60 | claudin-1 |
| **Occludin** | NM_001205255 | ACAGGCCTGATGAATTGCCA (58.5) | GTGAAGGCACGTCCTGTGT (59.3) | 218 | 60 | occludin |
| **SR-B1** | NM_005505 | TGCACTATGCCCAGTACGTC (58.7) | TAGGCCTGAATGGCCTCCTT (60.3) | 148 | 60 | scavenger receptor class B type I |
| **CD81** | NM_004356 | ACCTCCTGTATCTGGAGCTGG (60.0) | TTGGCGATCTGGTCCTTGTTG (59.4) | 235 | 60 | Cluster of Differentiation 81 |
| **ApoE** | XM_005258867 | CGCTTTTGGGATTACCTGCG (58.8) | GGGGTCAGTTGTTCCTCCAG (59.8) | 158 | 60 | Apolipoprotein E |
| **miR-122** | NR_029667.1 | ACACTCCAGCTGGGTGGAGTGTGACAATCC (65.7) | TGGTGTCGTGGAGTCG (48.5) | 66 | 60 | MiroRNA 122 |
| **SEC14L2** | NM_012429 | GGGATCCTTTAAGAGGCGGG (55.9) | GTCATCTGGATTCGGCAGGG (55.9) | 262 | 60 | SEC14-like protein 2 |
| **GAPDH** | NG_009349.4 | GAAATCCCATCACCATCTTCC (55.0) | AAATGAGCCCCAGCCTTCTC (59.6) | 124 | 60 | glyceraldehyde-3-p dehydrogenase |

Sequence of the primers and the conditions used in quantitative real-time PCR (qPCR).
